# Influence of X-ray computed tomography (CT) exposure and reconstruction parameters on positron emission tomography (PET) quantitation

**DOI:** 10.1186/s40658-020-00331-w

**Published:** 2020-10-09

**Authors:** Ivan Ho Shon, Christopher Reece, Thomas Hennessy, Megan Horsfield, Bruce McBride

**Affiliations:** 1 Department of Nuclear Medicine and PET, Prince of Wales Hospital, Level 2 Campus Centre, Barker Rd, Randwick, 2031 NSW Australia; 2 Prince of Wales Clinical School, UNSW Medicine, Kensington, NSW 2025 Australia; 3 Centenary Institute of Cancer Medicine and Cell Biology, University of Sydney, Missenden Rd, Camperdown, NSW 2050 Australia

**Keywords:** Positron-emission tomography, Tomography, X-ray computed, Positron emission tomography computed tomography

## Abstract

**Background:**

The CT of PET CT provides diagnostic information, anatomic localisation and attenuation correction (AC). When only AC is required, very lose dose CT is desirable. CT iterative reconstruction (IR) improves image quality with lower exposures however there is little data on very low dose IR CT for AC of PET. This work assesses the impact of CT exposure and reconstruction algorithm on PET voxel values.

**Method:**

An anthropomorphic torso phantom was filled with physiologically typical [18]F concentrations in heart, liver and background compartments. A 17-mm-diameter right lung “tumour” filled with [18]F was included (surrounding lung contained no 18[F]). PET was acquired followed by 24 CT acquisitions with varying CT exposures (15–50 mAs, 80–120 kVp, pitch 0.671 or 0.828). Each CT was reconstructed twice using filtered back projection (FBP) or IR and these used for AC of PET. The reference PET reconstruction (RR) used CT acquired at 50 mAs, 120 kVp, pitch 0.828, IR, all others were test PET reconstructions (TR). Regions of interest (ROIs) were drawn in the liver, soft tissue and over “tumour” on each TR and compared with the RR. Voxel values in each TR were compared to the RR using a paired *t* test and by calculating which and what proportion of voxels in each TR differed by a quantitatively significant difference (QSD) from the RR.

**Results:**

TRs reconstructed using lower dose CTs underestimated mean and maximum ROI activity relative to the RR; greater with IR than FBP. Once CT dose index (CTDI) increased to 1 mGy, differences were less than QSD. On voxel analysis, all TRs were significantly different to the RR (*p* < 0.0001). TRs reconstructed at the lowest CT exposure with IR had 6% of voxels that differed by greater than QSD. Differences were reduced with increasing CTDI and FBP reconstruction. Voxels which exceeded the QSD were spatially localised to regions of high activity, interfaces between different attenuation and areas of CT beam hardening.

**Conclusions:**

Very low dose CT exposures are feasible for accurate PET AC. Scanner- and reconstruction-specific validation should be employed prior very low dose CT AC for PET.

## Background

Positron emission tomography (PET) is a routine imaging modality with wide clinical applications especially in oncology particularly with 2-fluoro-2-deoxyglucose (FDG). Currently, clinical PET scanners are combined with X-ray computed tomography (CT) scanners. PET CT scanners have proved to be a revolution by combining molecular imaging provided by PET with diagnostic anatomic information, anatomic localisation and an attenuation correction (AC) map provided by CT [1, 2]. However, the CT component of PET CT examinations may not necessarily be required for all of these purposes in all examinations and X-ray exposure should be optimised for the indication(s) for which the CT component is being performed—higher exposures are required for diagnosis and much lower exposures may be used for generation of AC maps. While much lower radiation exposures are needed to generate CT based AC maps, it is essential that the AC maps must remain accurate to enable accurate PET reconstruction and quantitation.

Traditionally, CT has been reconstructed with filtered back projection (FBP) techniques. More recently, iterative reconstruction (IR) algorithms have been used for CT reconstruction which are reported to result in better noise reduction, improved image quality and lower radiation dose in a variety of situations (including CT coronary angiography, abdominal and thoracic imaging) [3]. The ability of IR to reduce radiation exposures while maintaining image quality for diagnostic CT raises the question about whether IR would also enable further reductions in the required exposures for generation of AC maps for PET reconstruction. While the reductions in CT exposure may be small for routine clinical situations, there are specific situations where minimising exposures from CT performed for AC would be especially beneficial when frequent serial PET scans are being performed such as for biodistribution and dosimetric studies for development of novel positron emitting radiopharmaceuticals or for respiratory or cardiac gated studies. In these circumstances even small reductions in radiation exposure may be cumulatively significant.

Therefore the aim of this work is two-fold—firstly, to determine the minimum CT acquisition parameters needed for generation of AC maps and secondly, to determine if IR enables further reductions in radiation exposures while maintaining the accuracy of AC for PET reconstruction.

## Methods

### Phantom

**Fig. 1:**
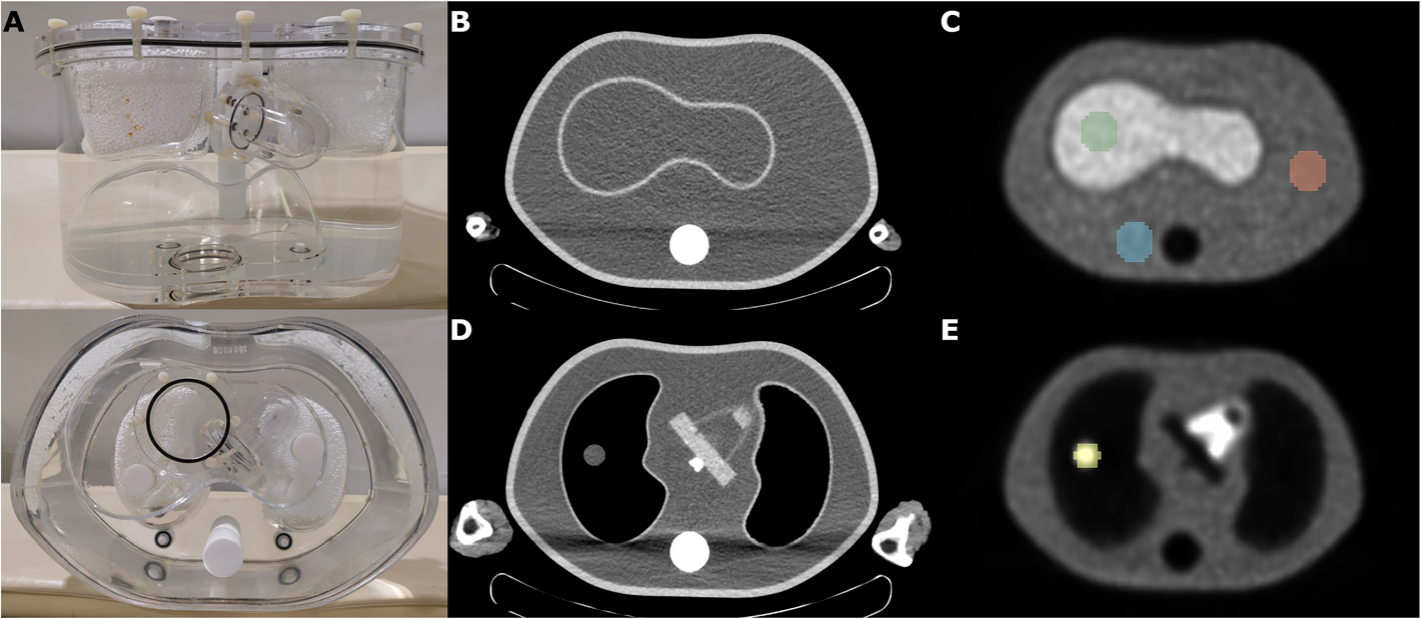
The DataSpectrum anthropomorphic phantom (lamb shanks not shown and the lung “tumour” is obscured by the polystyrene in the lung cavities) (**a**). Axial CT slices of the DataSpectrum anthropomorphic phantom, with adjacent lamb shanks and custom lung “tumour” insert (**b**, **d**) and corresponding axial slices of the reconstructed PET (**c**, **e**) with ROIs for the liver (green), soft tissue (orange), soft tissue in an area of CT beam hardening (blue) and the lung “tumour” (yellow). ROIs were copied to ensure consistent placement and no overlap into adjoining regions

An anthropomorphic torso phantom (DataSpectrum, Durham, NC, USA) was custom modified to include a 17-mm spherical lesion in the right lung. The soft tissue, liver and heart components of the phantom were filled with physiologically typical [18]F FDG concentrations, as was a 17 mm diameter spherical lesion in the right lung (Fig. 1). The phantom was filled such that soft tissue had a mean standard uptake value (SUV) of 1.0, the heart mean SUV of 4.0, liver mean SUV of 2.5 and “tumour” mean SUV of 4.0. No activity was filled into the lungs. To simulate the impact of attenuation from a patient’s arms, lamb shanks were placed on either side of the phantom. An emission PET scan was performed on a Philips Ingenuity TF 128 PET scanner (Philips Medical Systems, Cleveland) (2.5 min per bed position for 3 bed positions) followed by consecutive CT acquisitions at 15, 25, 40 and 50 mAs, 80, 100 and 120 kVp and pitch of 0.671 or 0.828, yielding a total of 24 CT acquisitions. Each of the CT acquisitions was then reconstructed with either FBP or IR (iDose level 3, Philips Medical System). This then yielded a total of 48 CT datasets which were then used to generate AC maps for reconstruction of the single PET acquisition. The reference PET dataset was reconstructed using the CT AC map derived from the CT acquisition acquired at 50 mAs, 120 kVp, pitch 0.828 and reconstructed with IR. PET reconstruction was performed using Philips Astonish TF, a list mode fully 3D iterative ordered subset expectation maximisation algorithm with “Blob” basis function ( 3 iterations, 3 subsets, kernel width = 18.1 cm, relaxation parameter = 1). PET data are corrected for decay, random coincidences (using the delayed window method), scatter (using the single scatter simulation approximation method) and point spread function effects. This was defined as the reference reconstruction (RR) as it is the current institutional standard clinical protocol for an average patient, is recommended by the manufacturer and is routinely validated as quantitatively accurate based on regular clinical validation phantom studies according to manufacturer recommendations. All other PET datasets reconstructed using the other combinations of CT acquisition and reconstruction parameters for AC (and using the same PET reconstruction algorithm as above) are termed test reconstructions (TRs). The CT dose index (CTDI) for each CT acquisition was recorded as reported by the acquisition workstation.

### Data analysis

#### Region of interest analysis

Two-dimensional circular regions of interest (ROIs) were drawn on the soft tissue, liver, lung lesion and on soft tissue where there was evidence of significant beam hardening artefact and the mean, maximum and standard deviation (SD) of SUV determined for each of these regions. Values were compared to the values obtained from the reference PET construction and compared with CTDI. To assess image noise, the SUV coefficient of variation (COV) was calculated using:

$$ \mathrm{SUV}\;\mathrm{COV}=\frac{\mathrm{SUV}\;\mathrm{SD}}{\mathrm{SUV}\;\mathrm{mean}}\times 100 $$

The difference in COV for each ROI in each FBP reconstructed TR compared with the ROI in the matching IR reconstructed TR was calculated as follows:

$$ \Delta \mathrm{COV}=\mathrm{SUV}\;{\mathrm{COV}}_{\mathrm{ROI}\;\mathrm{in}\;\mathrm{FBR}}\hbox{-} \mathrm{SUV}\;{\mathrm{COV}}_{\mathrm{ROI}\;\mathrm{in}\;\mathrm{IR}} $$

The range, mean, median and SD for the ΔCOV for all ROIs was calculated and the COVs for all ROIs in the FBP reconstructed datasets was compared to the matching ROIs in the IR reconstructed datasets using a two sided paired *t* test, with a confidence interval of 95% and the null hypothesis of equivalence.

#### Voxel analysis

Voxel-based analysis was undertaken to compare every voxel in the entire volume of the TRs to its matching voxel in the RR using R [4–6]. The RR and TR PET datasets were imported into R [7]. Initially, voxel data was compared between the TRs and the RR using a two-sided paired *t* test with a 95% confidence interval and the null hypothesis being equivalence.

Each voxel in each TR was compared with the matching voxel in the RR and the difference determined. To assess if the observed differences are potentially quantitatively and diagnostically significant, a quantitatively significant difference (QSD) was defined as one SD of the mean of the ROI drawn over the liver (as used for the ROI analysis described above).

QSD = SD_(Liver ROI of RR)_

For each TR, the proportion of all voxels that differed by greater than the QSD from the corresponding voxel in the RR was calculated.

In order to determine if the voxels that differed by QSD were randomly or non-randomly distributed, a volumetric mask was created containing only those voxels in the test PET dataset that were significantly different from the matching voxel in the reference PET dataset. This volumetric mask was then overlaid on the corresponding TR and representative orthogonal slices displayed [7].

## Results

### Acquisitions and CTDI

The 24 different combinations of CT acquisition parameters together with CTDI for each are shown in Table 1.

**Table 1:** CT acquisition parameter combinations used (reference CT reconstruction shown in bold)

Current (mAs)	Voltage (kVp)	Pitch	CTDI (mGy)
15	80	0.671	0.3
15	80	0.828	0.3
15	100	0.671	0.5
15	100	0.828	0.5
15	120	0.671	0.6
15	120	0.828	0.6
25	80	0.671	0.8
25	80	0.828	0.8
25	100	0.671	0.9
25	100	0.828	0.9
25	120	0.671	1
25	120	0.828	1
40	80	0.671	1
40	80	0.828	1
40	100	0.671	1.6
40	100	0.828	1.6
40	120	0.671	1.6
40	120	0.828	1.6
50	80	0.671	2
50	80	0.828	2
50	100	0.671	2.6
50	100	0.828	2.6
50	120	0.671	3.3
**50**	**120**	**0.828**	**3.3**

### Region of interest analysis

**Fig. 2:**
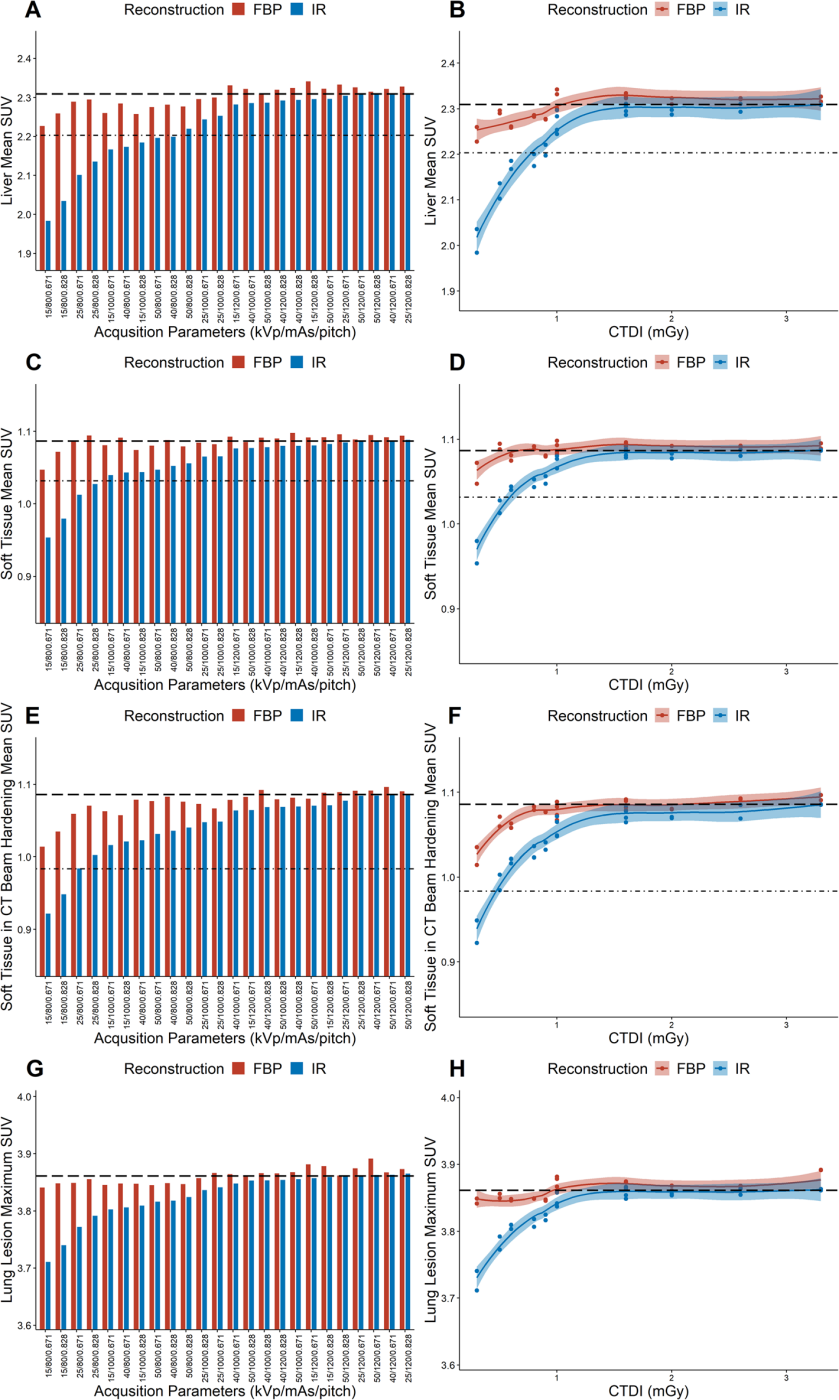
Mean SUV of liver (**a**), soft tissue (**c**), soft tissue in beam hardening region (**e**) and maximum SUV of the lung lesion (**g**) for each TR. The values from the RR are indicated by the dashed black line, and 1 standard deviation below the mean of the RR SUV is indicated by the dot dash black line (not applicable for the maximum SUV of the lung lesion). Mean SUV of liver (**b**), soft tissue (**d**), soft tissue in beam hardening region (f) and maximum SUV of the lung lesion (**h**) for each CT test reconstruction relative to CTDI. Shaded areas indicate confidence limits based on locally weighted regression fitting [22]

The ROI analysis demonstrated that both mean SUV for liver and soft tissue were underestimated at low CT exposure parameters. However, when CT was reconstructed using FBP irrespective of the CT exposure parameters, the mean SUV of ROIs in liver and soft tissue were within one SD of the mean SUV of the RR. However, IR of the CT resulted in much greater underestimation of the mean SUV for liver and soft tissue which at low CT exposures was greater than one SD of the mean SUV of the RR (specifically all acquisitions using 80 kVp and 100 kVp at 15 mAs). For the lung lesion the maximum SUV was assessed as the maximum SUV is the most used quantitative parameter for lesional analysis in clinical practice and to avoid partial volume effects. This demonstrated a similar trend with only a slight underestimation of lesional maximum SUV on CT reconstructed with FBP at low CT exposure parameters but a greater underestimation of lesional maximum SUV on CT reconstructed with IR at low CT exposure parameters (Fig. 2).

When compared with delivered radiation, there is concordant pattern with greater underestimation of SUV at low CTDI when the CT is reconstructed with IR than with FBP. At CTDIs greater than approximately 1 mGy the estimated SUV is similar regardless of reconstruction method (Fig. 2).

**Fig. 3:**
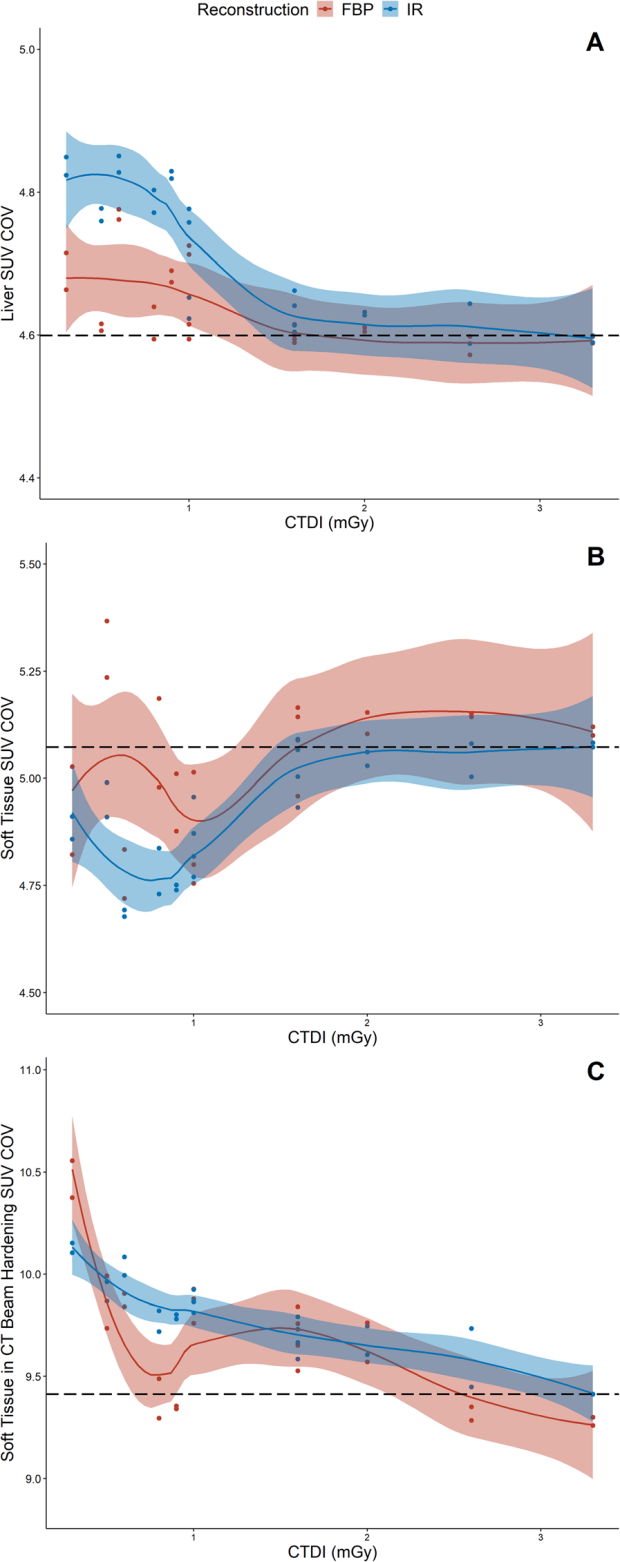
SUV COV compared to CTDI for each reconstruction of the ROIs in the liver (**a**), soft tissue (**b**) and soft tissue in CT beam hardening (**c**)

To assess the relationship between CT parameters and image noise, SUV COV was compared to CT exposure. At lower CT exposures, SUV COV was much more variable for reconstructions using either FBP or IR (Fig. 3). The SUV COV was significantly different for the ROIs of the liver, soft tissue and soft tissue in a region of CT beam hardening. However, while on average, SUV COV was higher for FBP in ROIs in soft tissue (i.e. ΔCOV positive), average SUV COV was greater for IR in ROIs in the liver and soft tissue in a region of beam hardening (i.e. ΔCOV negative) (Table 2).

**Table 2:** Minimum, median, mean and maximum Δ SUV COV for ROIs in FBP compared to IR reconstructed datasets and *p* values for comparison of paired SUV COVs

	Δ SUV COV				
	Minimum	Median	Mean	Maximum	*p* value
Liver	− 0.177	− 0.047	− 0.074	0.008	5.635e^−06^
Soft tissue	− 0.036	0.076	0.112	0.377	0.0001
Soft tissue in beam hardening	− 0.451	− 0.078	− 0.092	0.403	0.0473

### Voxel analysis

On individual voxel analysis using a paired *t* test, all TR’s differed significantly from the RR (*p* < 0.0001) (Table 3).

**Table 3:** Results of voxel-based analysis for each TR compared to the RR (*p* values stated as 0 are *p* values below that calculable by R)

mAs	kVp	Pitch	Reconstruction	CTDI	% Voxels >QSD	Maximum voxel difference	Mean voxel difference	*p* value
15	80	0.671	FBP	0.3	0.93%	23.03%	0.45%	0.00E+00
15	80	0.671	IR	0.3	5.96%	67.19%	1.43%	0.00E+00
15	80	0.828	FBP	0.3	0.31%	21.08%	0.35%	0.00E+00
15	80	0.828	IR	0.3	4.98%	56.79%	1.18%	0.00E+00
15	100	0.671	FBP	0.6	0.05%	15.40%	0.23%	0.00E+00
15	100	0.671	IR	0.6	1.22%	31.48%	0.56%	0.00E+00
15	100	0.828	FBP	0.6	0.07%	16.09%	0.23%	0.00E+00
15	100	0.828	IR	0.6	1.05%	29.29%	0.52%	0.00E+00
15	120	0.671	FBP	1	0.00%	5.85%	0.13%	0.00E+00
15	120	0.671	IR	1	0.00%	7.03%	0.14%	0.00E+00
15	120	0.828	FBP	1	0.00%	7.35%	0.14%	0.00E+00
15	120	0.828	IR	1	0.00%	6.38%	0.11%	0.00E+00
25	80	0.671	FBP	0.5	0.10%	14.62%	0.23%	0.00E+00
25	80	0.671	IR	0.5	3.24%	41.31%	0.88%	0.00E+00
25	80	0.828	FBP	0.5	0.05%	13.89%	0.20%	0.00E+00
25	80	0.828	IR	0.5	2.08%	38.10%	0.73%	0.00E+00
25	100	0.671	FBP	1	0.00%	6.91%	0.12%	0.00E+00
25	100	0.671	IR	1	0.19%	14.54%	0.28%	0.00E+00
25	100	0.828	FBP	1	0.00%	7.43%	0.12%	0.00E+00
25	100	0.828	IR	1	0.09%	14.38%	0.27%	0.00E+00
25	120	0.671	FBP	1.6	0.00%	4.06%	0.10%	0.00E+00
25	120	0.671	IR	1.6	0.00%	4.06%	0.08%	0.00E+00
25	120	0.828	FBP	1.6	0.00%	7.72%	0.09%	0.00E+00
25	120	0.828	IR	1.6	0.00%	7.19%	0.07%	0.00E+00
40	80	0.671	FBP	0.8	0.01%	11.50%	0.17%	0.00E+00
40	80	0.671	IR	0.8	1.22%	28.15%	0.55%	0.00E+00
40	80	0.828	FBP	0.8	0.00%	10.28%	0.16%	0.00E+00
40	80	0.828	IR	0.8	0.96%	22.87%	0.45%	0.00E+00
40	100	0.671	FBP	1.6	0.00%	4.43%	0.09%	0.00E+00
40	100	0.671	IR	1.6	0.00%	8.00%	0.15%	0.00E+00
40	100	0.828	FBP	1.6	0.00%	4.10%	0.08%	0.00E+00
40	100	0.828	IR	1.6	0.00%	6.22%	0.12%	0.00E+00
40	120	0.671	FBP	2.6	0.00%	4.59%	0.06%	0.00E+00
40	120	0.671	IR	2.6	0.00%	4.59%	0.05%	0.00E+00
40	120	0.828	FBP	2.6	0.00%	4.79%	0.06%	0.00E+00
40	120	0.828	IR	2.6	0.00%	6.30%	0.12%	0.00E+00
50	80	0.671	FBP	0.9	0.10%	13.61%	0.20%	0.00E+00
50	80	0.671	IR	0.9	1.13%	21.73%	0.50%	0.00E+00
50	80	0.828	FBP	0.9	0.03%	14.10%	0.19%	0.00E+00
50	80	0.828	IR	0.9	0.86%	18.32%	0.42%	0.00E+00
50	100	0.671	FBP	2	0.00%	4.59%	0.07%	2.94E-120
50	100	0.671	IR	2	0.00%	6.95%	0.11%	0.00E+00
50	100	0.828	FBP	2	0.00%	6.54%	0.11%	0.00E+00
50	100	0.828	IR	2	0.00%	7.07%	0.18%	0.00E+00
50	120	0.671	FBP	3.3	0.00%	5.93%	0.12%	0.00E+00
50	120	0.671	IR	3.3	0.00%	3.13%	0.04%	0.00E+00
50	120	0.828	FBP	3.3	0.00%	1.58%	0.03%	0.00E+00
50	120	0.828	IR	3.3	NA	NA	NA	NA

**Fig. 4:**
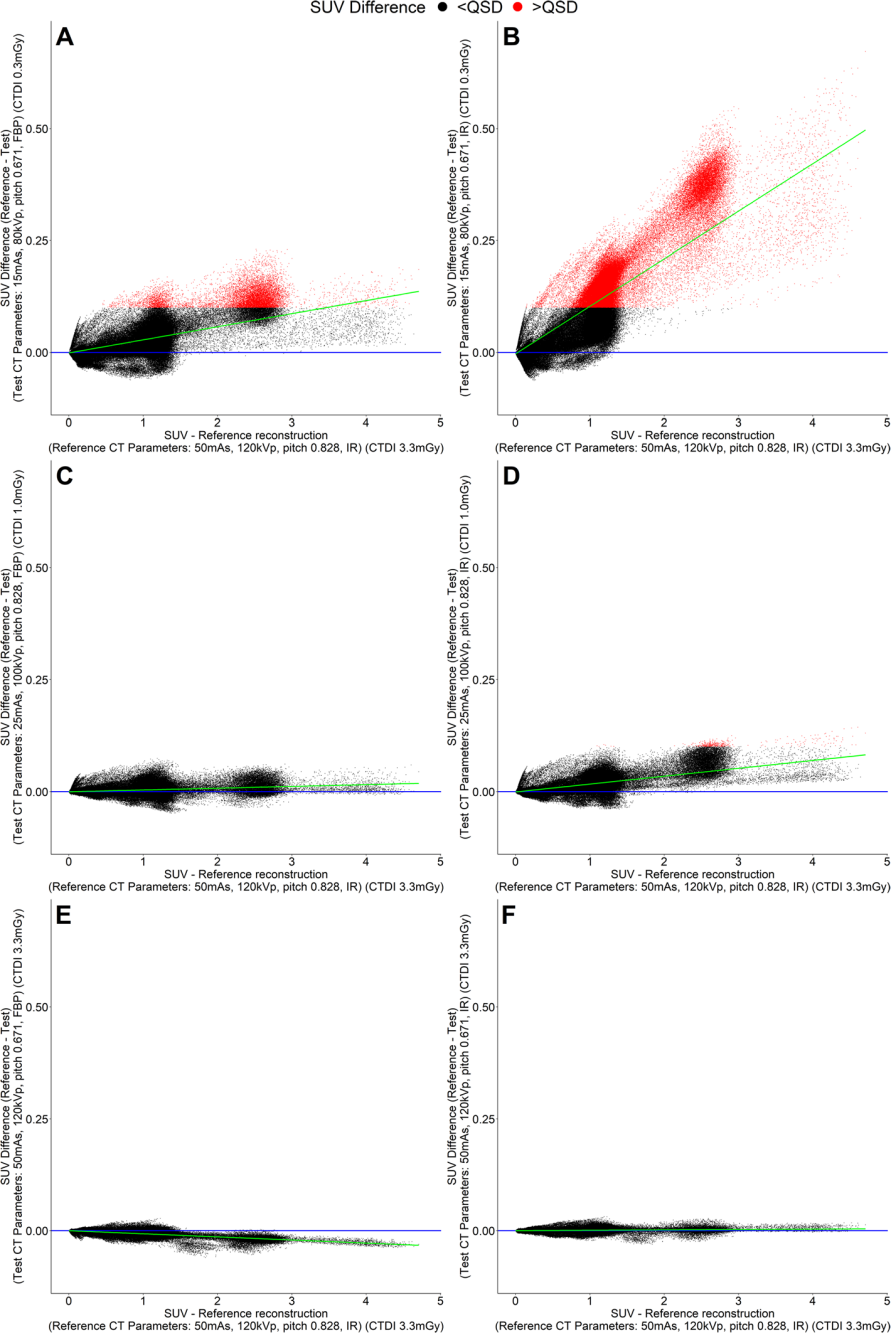
Graphs of voxel SUV differences between TRs and RR for individual voxels plotted against RR voxel SUV for 3 representative CT acquisition parameter combinations for low (CTDI 0.3 mGy) (**a**, **b**), medium (CTDI 1.0 mGy) (**c**, **d**) and high (CTDI 3.3 mGy) (**e**, **f**) X-ray exposure. CT reconstructed with filtered back projection (**a**, **c**, **e**) and iteratively (**b**, **d**, **f**). Each dot represents one voxel (black dots are voxels below QSD, and red dots are voxels greater than QSD). The green line is the linear regression fit for difference against reference value, and the blue line is the line of zero difference

Representative graphs of the difference between the SUV of each voxel in the TR compared to the RR relative to the voxel SUV in the RR are shown in Fig. 4. These demonstrate three findings. Firstly, that the greatest differences in voxel values are at the lowest CT exposures. Secondly, the differences are a systematic underestimation of voxel SUV in the TRs (i.e. RR–TR is greater than 0), especially in voxels with higher SUV. Lastly, the difference is much greater with IR than with FBP.

**Fig. 5:**
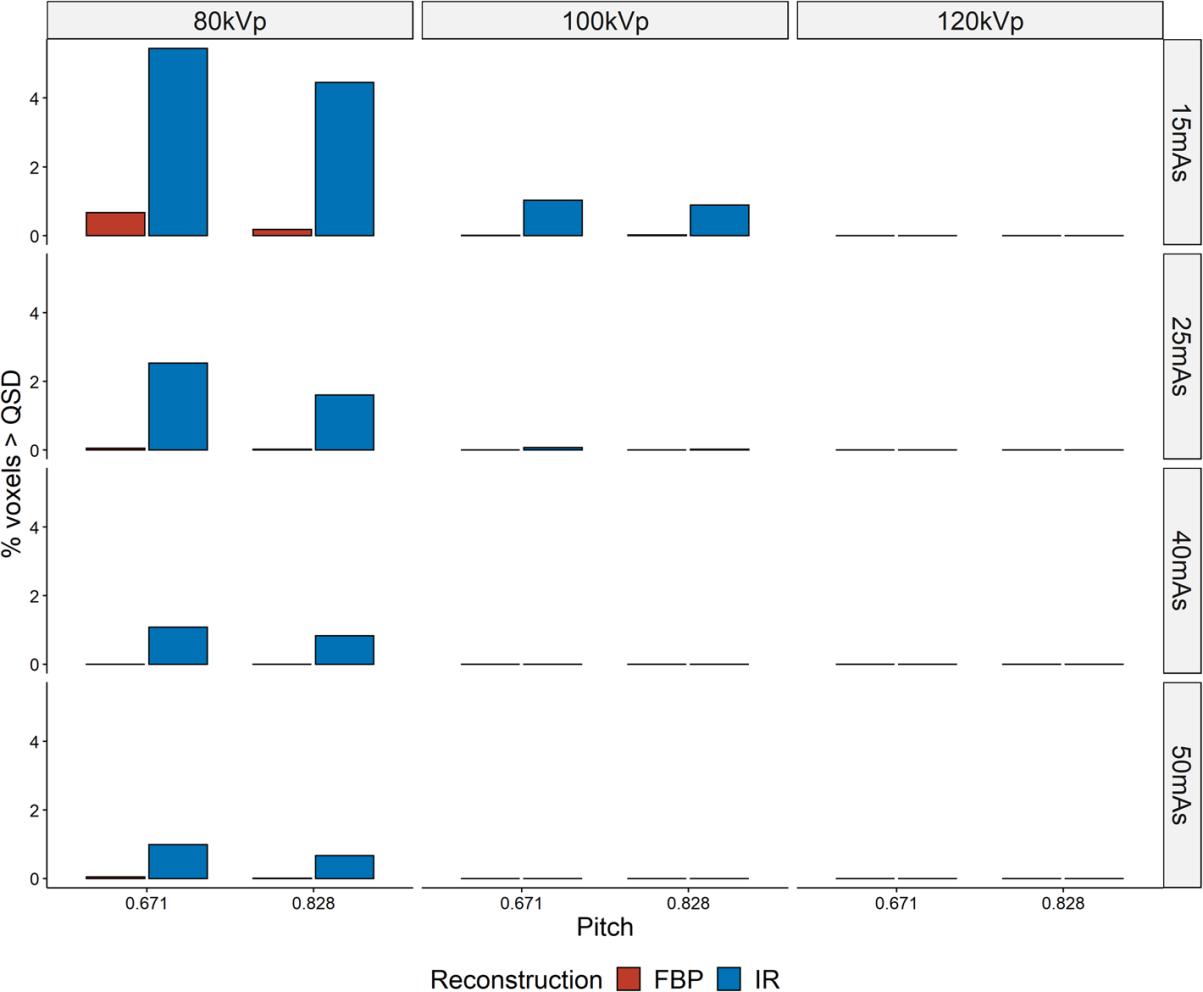
Proportion of voxels in TRs that differed from the RR by greater than QSD

The fraction of all voxels that differed by greater than the QSD for each TR is shown in Fig. 5. This demonstrates that with small increases in exposure and the use of FBP there is a rapid reduction in the number of voxels in the TR that exceed QSD. It also highlights that beam voltages of 80 kVp are insufficient to generate quantitatively accurate CT AC maps despite increasing beam current.

**Fig. 6:**
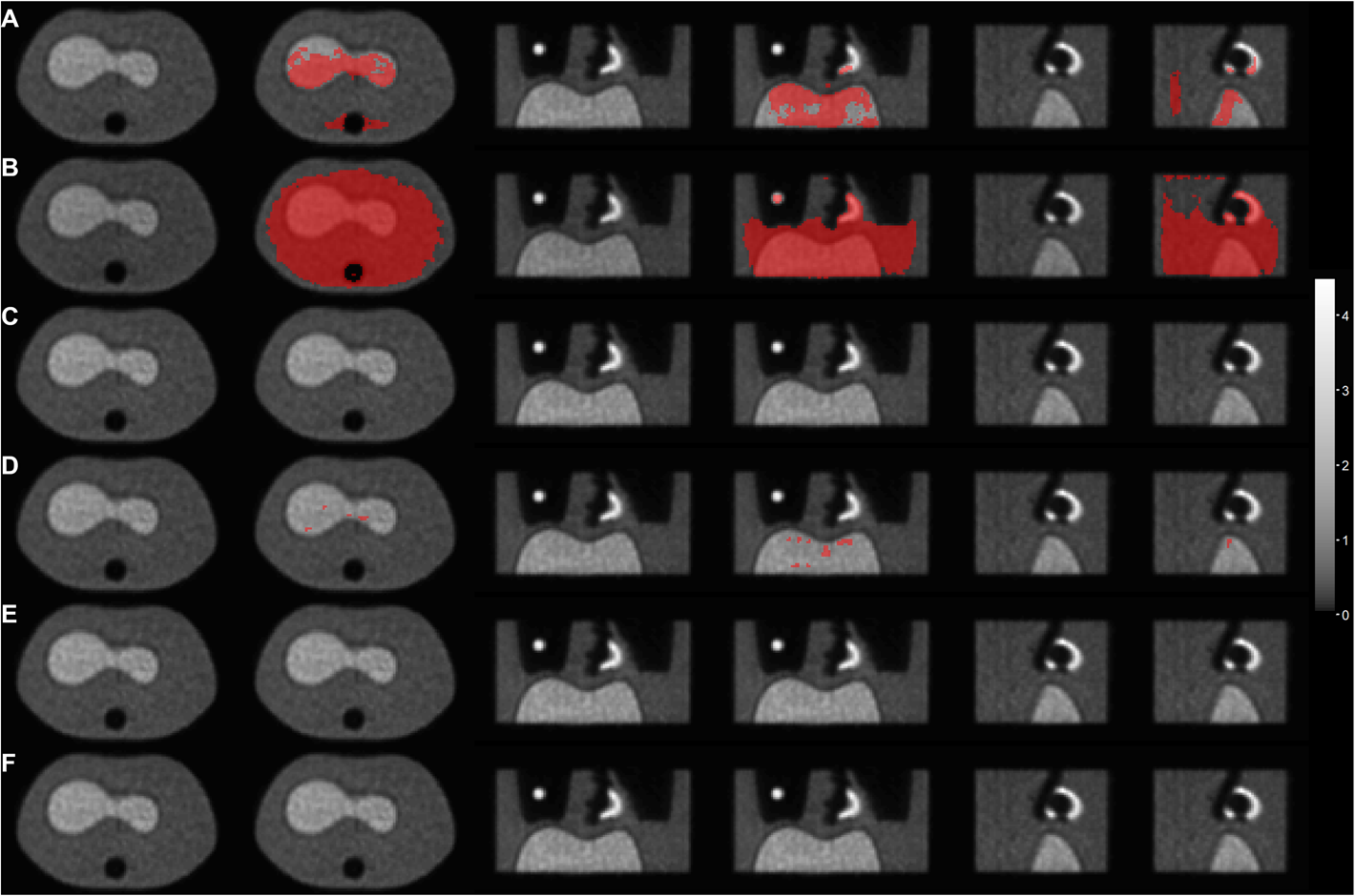
Representative axial, coronal and sagittal images of the phantom for 3 representative CT acquisition parameter combinations for low (CTDI 0.3 mGy) (**a**, **b**), medium (CTDI 1.0 mGy) (C,D) and high (CTDI 3.3 mGy) (**e**, **f**) X-ray exposure. CT reconstructed with FBP (**a**, **c**, **e**) and iteratively (**b**, **d**, **f**). The grayscale images are from the TR reconstruction (scale SUV 0–4.5). The adjacent image shows those voxels in the TR that differ by greater than the QSD from the RR overlaid in red

Qualitative image quality and spatial assessment of voxel differences are shown in Fig. 6. This demonstrates there is no qualitative difference in image quality between the various reconstructions and that those voxels where there is a QSD between TR and RR are spatially localised especially in areas of high activity, where there are changes in CT density (e.g. air to soft tissue interfaces), and in regions of prominent CT artefacts (such as associated with CT beam hardening in line with the spine and adjacent “limbs”).

## Discussion

PET is a widely adopted molecular imaging modality, being sensitive, inherently quantitative and able to image a wide range of molecular processes. It has important established clinical and research applications. In oncology, particularly using 2-fluoro-2-deoxyglucose (FDG), it has proven application for diagnosis, staging, treatment response and detection of relapse which results in significant changes in disease staging, treatment modality and intent [8, 9]. In addition to FDG, there are now a wide range of other PET radiopharmaceuticals in clinical and research use. Quantitation in PET is of particular importance as there is increasing evidence that quantitative parameters predict outcome [10] and is essential for biodistribution and dosimetry calculations for both clinical and research applications [11, 12].

Accurate AC is fundamental to high-quality, highly sensitive and accurate quantitative PET imaging. Initially, AC for PET was performed with sealed line sources [13]; however, approximately 20 years ago, Beyer and colleagues described development of the first combined PET CT scanner, using the CT component for generation of attenuation maps for PET reconstruction [1]. Combined PET CT is now widely accepted as superior to PET alone [2] and is now standard of care for PET imaging. In addition to providing AC, the CT component of the PET CT study may be used for anatomic localisation or diagnosis. Current practice guidelines recommend selecting CT acquisition parameters depending on the intended use, with recommendations for reduced voltage and/or current when the CT is used for AC only and with higher exposures required when CT is also used for diagnosis [14]. In most clinical and research purposes, in addition to AC, the CT component of a PET/CT study will also be used for anatomic localisation or diagnosis. However, in select circumstances, the CT component may only be required for AC, particularly when multiple repeated AC CTs are required over a short period of time. One example of this is for gated studies where an AC CT is required for each phase of the gated study. Another situation where CT may only be performed for AC is early phase human biodistribution and dosimetry studies where PET/CT scans are performed repeatedly in the same subject over a short timeframe, typically hours [12, 15]. In this setting, accurate AC maps are required for accurate quantitation; however, in the short interval between scans, no anatomic change would be expected and thus the CT component would serve no other purpose. In these settings, the repeated acquisition of CT may contribute more to the overall patient radiation exposure than the injected radiopharmaceutical, and thus it is essential that radiation exposure from the CT component be minimised according to the ALARA principle. This study was undertaken to establish the minimum CT exposures and optimise reconstruction parameters for accurate biodistribution and dosimetric assessment in preparation for a first in human study of a novel PET radiopharmaceutical for imaging cell death [16].

There is limited data regarding optimisation of the CT component of the PET CT acquisition. Recently, Bertolino and colleagues undertook a systematic review of CT protocols performed within a PET CT scan. Their rationale for undertaking this was the observation that unlike the PET acquisition, there is a lack of robust scientific literature regarding the optimisation of CT protocols used in PET CT. They concluded that dose is heavily dependent on the protocol intent (AC, anatomic localisation, or diagnosis). They did not conclude on specific parameters for CT acquisition within a PET CT rather suggesting periodic quality control considering technological advances [17]. There is very little data regarding dose optimisation of CT performed for AC. Faye and colleagues used five different anthropomorphic phantoms (newborn to medium adult) to assess the impact of acquisition parameters on CT image noise and adequacy of PET AC. They reported that significant dose reductions could be achieved, reporting that in paediatric patients adequate AC could be obtained with very low dose and with only an increase in tube voltages required to prevent under correction in adults. There are several differences between this previous study and this study. Firstly, Faye and colleagues assessed the adequacy of AC for PET quantitation qualitatively by visual inspection of the images—no quantitative analysis was performed on the reconstructed PET images (although this was undertaken on the CT AC map). Secondly, the study was performed on a PET CT scanner without capability for IR of CT [18].

Brady and Shulkin undertook a phantom and retrospective patient study to assess ultralow dose CT protocols reconstructed using adaptive statistical iterative reconstruction (ASIR) on PET and CT image quality and quantitation. With this protocol, they reported no change in SUV, background uniformity or spatial resolution of PET with up to 90% dose reduction and that there was an average deviation of only 2% for all cylindrical/spherical target lesions. In contrast to the current study, regions of interest were not considered outside of the target lesion (beyond background uniformity) and the scanner was from a different manufacturer with a different IR algorithm [19].

The paucity of published literature, and the absence of any specific data related to equipment at this institution or the specific application of quantitative imaging for first in human biodistribution, radiation dosimetry calculation and imaging, was the impetus for undertaking this study. With the intent of doing whole-body biodistribution studies, accurate quantitation at all sites (not just lesional sites) is essential and hence the region of interest analysis assessed both lesional and non-lesional regions. Voxel analysis of the reconstructed PET datasets were similarly undertaken to provide the broadest insight into subtle quantitative changes throughout the study.

In this study, determining what level of change to regard as significant is difficult and contentious and is dependent on many factors including technical, biologic and physical [20]. Both proportional and fixed changes were considered, with advantages and disadvantages to both approaches. Using a proportional change in activity relative to the concentration in the ROI may be appropriate as SD is higher with higher activity concentrations. However, proportional changes are also problematic as at lower activity concentrations proportional changes deemed significant may be of such a small absolute magnitude that they are not relevant in terms of diagnostic and dosimetric quantitation. At high activity concentrations, relatively small proportional changes below the level deemed significant may still have significant impact on clinical and research quantitation. Ultimately a fixed threshold change equivalent to 1 SD of SUV measured in the ROI within the liver was deemed significant, which equates to a change in SUV of 0.1 or ~4% of the mean SUV of the liver. It is acknowledged that this is a small change and in isolation would not be regarded as significant. However, to enable accurate comparison between studies whether performed for clinical indications (such as for assessment of treatment response following commencement of therapy) or for biodistribution and dosimetry calculations, Boellard described a wide range of factors which can affect PET quantification and stresses the importance of standardisation to minimise variability and improve accuracy of quantification. In particular, Boellard identified reconstruction parameters has a potential source of variability of up to 30% [20]. In defining the PERCIST 1.0 criteria, Wahl and colleagues use the SD of uptake within the liver in the formula for calculations both before and following treatment, and in addition state that liver SUV should generally be within 0.3 from study to study and much of this variability will be accounted for by biologic factors. Hence, an SUV change of 0.1 is approximately 30% of the expected interstudy reproducibility of mean liver uptake [21]. Given that this study was used to define parameters for multiple time point scanning for a first in human study, it was considered particularly important that a level of significance be set that was consistent with established clinical and research standards such as defined in PERCIST 1.0.

Both ROI and individual voxel analysis demonstrates that at very low CT exposures there is a systematic underestimate of mean SUV in all ROIs, which is greater when IR is used compared to FBP. With increasing CT exposure mean SUV in ROIs converges to the mean SUV obtained from the RR. When CTDI reaches 1 mGy, the difference is insignificant between TR and RR irrespective of whether IR or FBP are used on both ROI and voxel analysis. Based on ROI analysis, but not voxel analysis, CTDIs less than 1 mGy still result in differences of mean ROI SUV less than QSD between TRs and RR when reconstructed with FBP. However, as this study was undertaken as a prelude to a quantitative first in human biodistribution study, it was considered that both ROI and voxel analysis should not differ significantly between the RR and TR. Based on this, CT parameters chosen for AC for PET for the first in human study was 25 mAs, 100 kVp, pitch 0.828, FBP which delivers a CTDI of 1 mGy. Compared to the RR CTDI of 3.3 mGy, this represents an approximately 70% dose reduction.

In CT scans performed for diagnosis, IR has been reported to result in improved image quality while reducing CT dose; however, unexpectedly, it was observed that at the lower CT exposures IR of the CT used for AC of PET resulted in greater underestimation of activity compared to when FBP CT reconstruction was used for AC of PET. This is contradictory to that observed by Brady and Shulkin who also observed no change in image noise [19]. In this study, image noise expressed as SUV COV was much more variable at low CT exposures; in some regions, SUV COV was greater with IR and in other regions SUV COV was greater with FBP. SUV COV was similar at higher CT exposures regardless of reconstruction algorithm. Given this variability, it is unlikely that image noise alone is the explanation for the greater SUV underestimation with IR. Other possible explanations include differences in the IR algorithms and subsequent generation of segmented CT AC maps. The PET CT scanner used in this work has the option for differing levels of noise suppression (iDose levels) applied to IR and further work is warranted to investigate these. However, this study has demonstrated a 70% reduction in CTDI is possible without compromising quantitative accuracy. Further investigation of different iDose levels would be beneficial to see if further dose reductions are possible; however, the further gains would be relatively modest. Additionally, while use of IR at very low dose CT exposures may potentially enable better CT image quality, given the very low CT exposure even if further image quality improvements can be made it is unlikely the improvement will enable more than anatomic localisation which can already be adequately achieved with FBP CT images. The differences between this study and Brady and Shulkin’s study highlight the need for scanner- and reconstruction-specific assessment and periodic quality control audit as suggested by Bertolini and colleagues [17].

In conclusion, this study demonstrates the impact of CT acquisition parameters and reconstruction algorithms on AC for PET reconstruction and identifies appropriate parameters and algorithms to minimise exposure when CT is performed only for AC of PET studies on the Philips Ingenuity TF scanner. More generally, it demonstrates a method for assessment of the impact of CT acquisition parameters and reconstruction algorithms on quantitative accuracy of PET reconstructions that is broadly applicable to all PET CT scanners to enable scanner- and reconstruction-specific CT dose optimisation.

## Data Availability

The datasets used and/or analysed during the current study are available from the corresponding author on reasonable request.

## Abbreviations

CT: X-ray computed tomography
PET: Positron emission tomography
AC: Attenuation correction
FBP: Filtered back projection
IR: Iterative reconstruction
TR: Test reconstruction
RR: Reference reconstruction
QSD: Quantitatively significant difference
ROI: Region of interest
CTDI: CT dose index
SD: Standard deviation
COV: Coefficient of variation
